# Clinical performance and head-to-head comparison of CSF p-tau235 with p-tau181, p-tau217 and p-tau231 in two memory clinic cohorts

**DOI:** 10.1186/s13195-023-01201-0

**Published:** 2023-03-10

**Authors:** Juan Lantero-Rodriguez, Agathe Vrillon, Aida Fernández-Lebrero, Paula Ortiz-Romero, Anniina Snellman, Laia Montoliu-Gaya, Wagner S. Brum, Emmanuel Cognat, Julien Dumurgier, Albert Puig-Pijoan, Irene Navalpotro-Gómez, Greta García-Escobar, Thomas K. Karikari, Eugeen Vanmechelen, Nicholas J. Ashton, Henrik Zetterberg, Marc Suárez-Calvet, Claire Paquet, Kaj Blennow

**Affiliations:** 1 Department of Psychiatry & Neurochemistry, Institute of Neuroscience and Physiology, the Sahlgrenska Academy at the University of Gothenburg, Gothenburg, Sweden; 2 Institut national de la santé et de la recherche médicale U1144 Optimisation Thérapeutique en Neuropsychopharmacologie, Université de Paris, Paris, France; 3 Centre de Neurologie Cognitive, Groupe Hospitalo Universitaire Assistance Publique Hôpitaux de Paris Nord Hôpital Lariboisière Fernand-Widal, Paris, France; 4 Barcelonaβeta Brain Research Center (BBRC), Pasqual Maragall Foundation, Barcelona, Spain; 5 IMIM (Hospital del Mar Medical Research Institute), Barcelona, Spain; 6 Cognitive Decline and Movement Disorders Unit, Neurology Department, Hospital del Mar, Barcelona, Spain; 7 Turku PET Centre, University of Turku, Turku University Hospital, Turku, Finland; 8 Graduate Program in Biological Sciences: Biochemistry, Universidade Federal do Rio Grande do Sul (UFRGS), Porto Alegre, Brazil; 9 Department of Medicine, Universitat Autònoma de Barcelona, Barcelona, Spain; 10 Department of Psychiatry, School of Medicine, University of Pittsburgh, Pittsburgh, PA USA; 11 ADx NeuroSciences, Technologiepark 94, Ghent, Belgium; 12 Centre for Age-Related Medicine, Stavanger University Hospital, Stavanger, Norway; 13 Department of Old Age Psychiatry, Maurice Wohl Clinical Neuroscience Institute, King’s College London, London, UK; 14 NIHR Biomedical Research Centre for Mental Health & Biomedical Research Unit for Dementia at South London & Maudsley NHS Foundation, London, UK; 15 Clinical Neurochemistry Laboratory, Sahlgrenska University Hospital, Mölndal, Sweden; 16 Department of Neurodegenerative Disease, Queen Square Institute of Neurology, University College London, London, UK; 17 UK Dementia Research Institute, University College London, London, UK; 18 Hong Kong Center for Neurodegenerative Diseases, Hong Kong, China; 19 Wisconsin Alzheimer’s Disease Research Center, University of Wisconsin School of Medicine and Public Health, University of Wisconsin-Madison, Madison, WI USA; 20 Centro de Investigación Biomédica en Red de Fragilidad y Envejecimiento Saludable (CIBERFES), Madrid, Spain

**Keywords:** Alzheimer’s disease, CSF, Biomarkers, p-tau235, p-tau181, p-tau217, p-tau231, Memory clinic

## Abstract

**Background:**

Cerebrospinal fluid (CSF) p-tau235 is a novel biomarker highly specific of Alzheimer’s disease (AD). However, CSF p-tau235 has only been studied in well-characterized research cohorts, which do not fully reflect the patient landscape found in clinical settings. Therefore, in this multicentre study, we investigated the performance of CSF p-tau235 to detect symptomatic AD in clinical settings and compared it with CSF p-tau181, p-tau217 and p-tau231.

**Methods:**

CSF p-tau235 was measured using an in-house single molecule array (Simoa) assay in two independent memory clinic cohorts: Paris cohort (Lariboisière Fernand-Widal University Hospital Paris, France; *n*=212) and BIODEGMAR cohort (Hospital del Mar, Barcelona, Spain; *n*=175). Patients were classified by the syndromic diagnosis (cognitively unimpaired [CU], mild cognitive impairment [MCI] or dementia) and their biological diagnosis (amyloid-beta [Aβ]+ or Aβ $-$). Both cohorts included detailed cognitive assessments and CSF biomarker measurements (clinically validated core AD biomarkers [Lumipulse CSF Aβ_1–42/40_ ratio, p-tau181 and t-tau] and in-house developed Simoa CSF p-tau181, p-tau217 and p-tau231).

**Results:**

High CSF p-tau235 levels were strongly associated with CSF amyloidosis regardless of the clinical diagnosis, being significantly increased in MCI Aβ+ and dementia Aβ+ when compared with all other Aβ− groups (Paris cohort: *P* ˂0.0001 for all; BIODEGMAR cohort: *P* ˂0.05 for all). CSF p-tau235 was pronouncedly increased in the A+T+ profile group compared with A−T− and A+T− groups (*P* ˂0.0001 for all). Moreover, CSF p-tau235 demonstrated high diagnostic accuracies identifying CSF amyloidosis in symptomatic cases (AUCs=0.86 to 0.96) and discriminating AT groups (AUCs=0.79 to 0.98). Overall, CSF p-tau235 showed similar performances to CSF p-tau181 and CSF p-tau231 when discriminating CSF amyloidosis in various scenarios, but lower than CSF p-tau217. Finally, CSF p-tau235 associated with global cognition and memory domain in both cohorts.

**Conclusions:**

CSF p-tau235 was increased with the presence of CSF amyloidosis in two independent memory clinic cohorts. CSF p-tau235 accurately identified AD in both MCI and dementia patients. Overall, the diagnostic performance of CSF p-tau235 was comparable to that of other CSF p-tau measurements, indicating its suitability to support a biomarker-based AD diagnosis in clinical settings.

**Supplementary Information:**

The online version contains supplementary material available at 10.1186/s13195-023-01201-0.

## Background

Alzheimer’s disease (AD) is characterized by the accumulation of aggregated amyloid-beta (Aβ) and hyperphosphorylated tau protein into extracellular Aβ plaques and intracellular neurofibrillary tangles (NFTs), respectively [1, 2]. These two immunohistochemical findings, together with gross atrophy and synaptic and neuronal loss, represent the hallmarks of AD and post-mortem examination demonstrating the presence of these proteinopathies is required for definitive diagnosis [3]. Validated imaging and fluid biomarkers increasingly support the clinical assessment and diagnosis of AD during life. Imaging biomarkers include positron emission tomography (PET) using radio-ligands capable of specifically binding Aβ and tau aggregates, whereas widely validated fluid biomarkers currently include Aβ_1–42_ (or Aβ_1–42/40_ ratio), total tau (t-tau) and phosphorylated tau at threonine 181 (p-tau181), all measured in cerebrospinal fluid (CSF) [4–7] often by full-automated instruments.

Recently, the field of fluid biomarkers in neurodegeneration has undergone a remarkable expansion, particularly with regard to biomarkers measuring different variants of tau protein. This has resulted in a wide range of novel p-tau biomarkers, measured with different methods and platforms (fundamentally immunoassays and mass spectrometry) [8–11]. Different p-tau residues may change in different stages of the Alzheimer’s continuum. For example, p-tau231 is the first p-tau residue increasing in preclinical AD, confirming the earliest underlying AD processes [11, 12] whereas p-tau217, with its pronounced fold changes and strong association with AD pathological hallmarks, appears to be the most suitable p-tau biomarker for AD diagnosis and patient monitoring [13, 14]. Moreover, different p-tau epitopes may provide different information about the disease and some of them be more suited as a state, stage, or prognostic biomarkers. The most recent p-tau biomarker to demonstrate potential utility in relation to AD is p-tau235 [15], a phosphorylation site that has been found to be a prominent feature in paired helical filaments (PHFs) [16–19]. Recently, using a targeted mass spectrometry method, our group has demonstrated that p-tau235 is highly increased in neuropathologically confirmed AD brain when compared with control cases [15]. These findings align with previous studies, indicating that phosphorylation at serine 235 hampers tubulin assembly, compromising normal microtubules dynamics [20, 21]. We developed a single molecule array (Simoa) assay capable of measuring p-tau235 in CSF, demonstrating that this novel biomarker is highly specific for AD and that it increases during preclinical stages, when only subtle abnormalities in CSF Aβ_1–42/40_ can be detected [15]. Interestingly, when examining post-mortem brain samples, phosphorylation at serine 235 appears to occur only when preceded by nearby phosphorylation at threonine 231, in what appears to be a sequential phosphorylation event. Due to the translatability of this sequential phosphorylation event from the brain into CSF, p-tau235 has been previously proposed as a potentially useful staging biomarker of preclinical AD (CSF p-tau235 positivity being indicative of late asymptomatic AD stage) [15]. With clinical trials increasingly focusing their intervention at the earliest stages of AD, CSF p-tau235 may be useful to discriminate early from late preclinical AD cases and to evaluate if novel compounds effectively prevent disease progression.

In order to further characterize this novel p-tau biomarker and contextualize its performance with other CSF p-tau species, we previously compared CSF p-tau235 with CSF p-tau217 and p-tau231 in a well-characterized research cohort including the AD *continuum* and a preclinical AD cohort. We demonstrated that CSF p-tau235 displays a statistically equal performance to that of CSF p-tau217 and p-tau231 when discriminating cognitively impaired CSF Aβ-positive cases from cognitively impaired CSF Aβ-negative cases. However, the cases enrolled in that study belonged to a well-characterized research cohort, which does not fully reflect the patients from daily clinical practice including atypical AD, other dementia and comorbidities. Therefore, evaluating the performance of CSF p-tau235 in the routine practice of a memory clinic is much needed, since in these settings patients present with a higher heterogeneity in demographics, comorbidities and disease presentations [22–25]. As a secondary aim, we tested whether CSF p-tau235 was associated with cognitive performance.

To this end, we measured this novel p-tau biomarker in two independent memory clinic cohorts, compared its performance with N-terminal directed Simoa immunoassays targeting CSF p-tau181, p-tau217 and p-tau231, and assessed its relation with cognitive status.

## Methods

### Study participants

#### Paris cohort

Paris cohort enrolled a total of 212 patients who had undergone CSF analysis at the Centre of Cognitive Neurology at Lariboisière Fernand-Widal University Hospital between March 2014 and December 2019, including participants with subjective cognitive decline (SCD, *n*=21), non-AD mild cognitive impairment (non-AD MCI, *n*=45), AD-MCI (*n*=40), AD dementia (*n*=75) and non-AD dementia (*n*=31). Non-AD dementia patients encompassed patients with dementia with Lewy bodies (DLB, *n*=12), frontotemporal dementia (FTD, *n*=15), vascular cognitive impairment and dementia (VCID, *n*=3) and Creutzfeldt Jakob disease (*n*=1). Patients underwent a thorough clinical examination involving personal medical and family histories, treatment, neurological examination, extensive neuropsychological assessment, APOE genotyping, brain magnetic resonance imaging (MRI) extensive neuropsychological evaluation, MRI, APOE genotyping, blood and CSF analysis and fluid sampling for collection (blood and CSF). The diagnosis for each patient was made during multidisciplinary consensus meetings (including neurologists, neuropsychologists, gerontologists, neuroradiologist and biochemists) considering results of validated CSF biomarkers and according to clinical diagnostic criteria for AD dementia [3], MCI due to AD (AD-MCI) [26], DLB [27] and FTD [28]. AD patients displayed CSF biomarkers on the AD *continuum* [3]. MCI of other causes (non-AD MCI**)** included patients with psychiatric disorder, sleep apnea, or systemic disease. Non-AD MCI presented with normal CSF biomarkers or suspected non-Alzheimer pathophysiology (normal Aβ_1-42/40_, high p-tau and/or high t-tau). Included SCD participants were individuals with several years of clinical follow-up for a clinical complaint, presenting with normal cognitive testing and no abnormalities at imaging and CSF examinations [29, 30].

#### BIODEGMAR cohort

The BIODEGMAR cohort is an observational longitudinal study that enrolls patients with neurodegenerative diseases visiting the Cognitive Decline and Movement Disorders Unit of Hospital del Mar (Barcelona, Spain). The procedures of the BIODEGMAR study include extensive neuropsychological evaluation, MRI, *APOE* ε4 genotyping, lumbar puncture for CSF collection and blood sampling. Clinical evaluation was performed by a neurologist, including anamnesis, physical examination and clinical diagnosis. Neuropsychological evaluation was performed by a neuropsychologist and consisted of a series of standardized cognitive tests and functional scales. A comprehensive description of the BIODEGMAR cohort has been previously published, with further details on the inclusion and exclusion criteria and on core AD CSF biomarker procedures, including assay details and cutoff determination [31, 32]. In this study, 175 patients of the BIODEGMAR cohort were included from 27 April 2017 to 24 July 2020. The clinical diagnoses included SCD (*n*=18, similar diagnostic criteria as for Paris cohort), MCI (*n*=74) and, for subjects with moderate to severe cognitive impairment, possible AD dementia (*n*=11), probable AD dementia (*n*=43), LBD (*n*=2), extrapyramidal syndrome (*n*=2), vascular cognitive impairment and dementia (VCID; *n*=4), progressive supranuclear palsy (PSP; *n*=2), corticobasal syndrome (*n*=4), behavioral variant of FTD (bvFTD; *n*=4), primary progressive aphasia (PPA; *n*=8) and cerebral amyloid angiopathy (CAA; *n*=3).

### Cohort stratification

Patient data from the Paris cohort and the BIODEGMAR cohort was examined using two stratification criteria: (i) according to clinical syndrome (cognitively unimpaired [CU], MCI, or dementia) and CSF amyloid status (Aβ−/Aβ+, as defined by Lumipulse CSF Aβ_1-42/40_, Supplementary Table 3), resulting in six groups: CU Aβ−, CU Aβ+, MCI Aβ−, MCI Aβ+, dementia Aβ− and dementia Aβ+ (clinical diagnosis included in each group are available in Supplementary Tables 1 and 2); (ii) based on the Aβ (A) and tau (T) status defined using CSF Aβ_1–42/40_ and p-tau181, respectively (Lumipulse®) into A−T−, A+T− and A+T+ (the A−T+ group considered suspected non-AD pathology [SNAP] was not included in the statistical analysis, but is depicted in the boxplots). The clinical diagnoses included in each group are available in Supplementary Tables 5 and 6). Additionally, CSF p-tau235 levels across clinical diagnostic groups for both cohorts are available in Supplementary Figure 1.

### Neuropsychological assessment

All included participants were evaluated using the Mini-Mental State Examination (MMSE). Detailed cognitive assessment was available for a subsample of participants in both the Paris cohort (total *n*=135: CU [*n*=14], MCI patients [*n*=64], dementia patients [*n*=57]) and the BIODEGMAR cohort (total *n*=139: CU [*n*=18], MCI [*n*=59], dementia [*n*=62]). In the Paris cohort, memory domain scores were evaluated using total immediate and delayed recall of Free and Cued Selective Reminding Test (FCSRT), and the delayed matching-to-samples 48 test (DMS 48) for visual memory testing. Executive function was assessed using forward and backward digit span, frontal assessment battery and letter and animal fluencies. The language domain was evaluated using the Dénomination Orale 80 test, a naming test. In BIODEGMAR cohort, memory domain was evaluated using total immediate and delayed recall of FCSRT and the Memory Impairment Screen; executive functions, with backward digit span, TMT A and B; language domain, with the Boston naming test. *Z*-scores were computed from the control group scores as a reference. Domain scores were obtained by averaging *z*-scores of the individual tests available results within that domain and the global cognition score by averaging the 3 domains’ *z*-scores.

### Biomarker measurements

In the Paris cohort, core AD CSF biomarkers (Aβ_1–42/40_ ratio, p-tau181 and t-tau) were measured using the clinically validated Lumipulse® G1200 assay (Fujirebio) [33]. Biomarker measurements for CSF p-tau181, p-tau217 and p-tau231 were performed using *in-house* Simoa assays. CSF p-tau181 and p-tau217 measurements were previously reported [34] and were included here in order to allow the comparison with novel CSF p-tau235. In the BIODEGMAR cohort, core AD CSF biomarkers (Aβ_1–42/40_ ratio, p-tau181 and t-tau) were measured at Laboratori de Referència de Catalunya (LRC) with Lumipulse G600II (Fujirebio). Cutoff values for biomarkers and their ratios (Aβ_1–42/40_, p-tau181, t-tau) were previously defined in the CORCOBIA study [31]. Biomarker measurements for CSF p-tau181 and p-tau231 were performed using *in-house* Simoa assays and were previously reported [32].

### CSF p-tau235 measurements

CSF p-tau235 measurements were performed at the Clinical Neurochemistry Laboratory at the University of Gothenburg using a Simoa HD-X instrument (Quanterix), and all samples were blinded. Prior to the analysis, samples were allowed to thaw at room temperature for 45 min. Thawed samples were subsequently vortexed for 15 s, after which they were plated and diluted 1:2 using Tau2.0 sample assay diluent (Quanterix). Due to sample volume availability, CSF samples from the Paris cohort were run in singlicates, while BIODEGMAR cohort samples were run in duplicates. An eight-point calibration curve was generated using recombinant full-length GSK-3β-phosphorylated tau-441 (SignalChem) and run in duplicates. Two internal quality controls (iQC, low and high) were run at the beginning and end of each plate, also in duplicates. Repeatability (%CV_r_) and intermediate precision (%CV_Rw_) values for both cohorts were ˂15%. Further details on assay specifications and validation can be found elsewhere [15].

### Statistical analysis

Statistics were performed using SPSS (v26, IBM, Armonk, NY, USA) and Scistat (Ostend, Belgium). Graphs were generated using GraphPad PRISM (v7.03, San Diego, CA, USA) and R version 4.1 (https://www.r-project.org/). Boxplots show the median and interquartile range (IQR), with individual data points for all participants always shown. Statistical analyses were performed using parametric or non-parametric methods when appropriate, based on the normality or not of the data. Group comparisons between two categories were performed using a *t*-test and Mann-Whitney *U* test. All comparisons of multiple groups were tested with a one-way ANOVA adjusted for age and sex followed by Bonferroni-corrected post-hoc pairwise comparisons. Adjusted *P-*values for covariates are indicated as *P*_*ADJ*_. For each of the CSF p-tau biomarkers, accuracy for binary outcomes was determined using receiver operating characteristics (ROC) analysis and area under the curve (AUC). AUC values are accompanied by the 95% confidence interval, abbreviated as CI_95%_. Performance comparison between different p-tau biomarkers was analysed using DeLong test (MedCalc). Spearman’s rank correlation (r_S_) was used to determine associations between biomarkers. Association of CSF p-tau biomarkers with global and domain-specific cognitive* z*-scores was studied with linear regression models adjusted for age, sex and level of education.

## Results

### Cohort characteristics

A total number of 387 participants were included in this study, which belong to two independent clinical cohorts (Paris cohort [*n*=212]; BIODEGMAR cohort [*n*=175]). Demographics and stratification in syndromic groups (CU Aβ−/Aβ+, MCI Aβ−/Aβ+ and dementia Aβ−/Aβ+) are presented in Table 1 for both cohorts. In the Paris cohort, MCI Aβ+ and dementia Aβ+ were older than CU Aβ− group (*P*<0.0001). There was a weak yet significant association with age in both cohorts (Paris cohort: *r*_S_=0.32, *P* <0.0001; BIODEGMAR cohort: *r*_S_ =0.17, *P* <0.05). There were no sex differences between groups (*P* ≥0.05; *χ*^2^ test). In both cohorts, CSF p-tau levels were significantly higher in *APOE* ε4 carriers (overall *P* <0.0001). As expected, there were significant differences in MMSE scores across groups in both cohorts (*P* <0.0001), which, as compared with CU, gradually decreased in MCI and further in patients with dementia.

**Table 1:** Demographics and biomarkers levels

	**Paris cohort (*n* = 212)**						**BIODEGMAR cohort (*n* =175 )**						
	**CU** **Aβ−**	**MCI** **Aβ−**	**Dementia Aβ−**	**MCI** **Aβ+**	**Dementia** **Aβ+**	***P*-value**	**CU** **Aβ−**	**MCI** **Aβ−**	**Dementia** **Aβ−**	**CU** **Aβ+**	**MCI** **Aβ+**	**Dementia Aβ+**	***P*-value**
	***n* = 21**	***n* = 47**	***n* = 27**	***n* = 40**	***n* = 77**		***n*=11**	***n*=28**	***n*=19**	***n*=7**	***n*=46**	***n*=64**	
**Age, years**	64.6 (9.42)	66.30 (9.58)	66.04 (7.73)	72.40 (7.96)^a^	71.90 (8.61)^a^	**< 0.0001**	70.4 (5.8)	71.4 (6.1)	70.0 (6.1)	70.1 (4.5)	74.4 (4.8)	73.5 (4.5)	**0.009**
**Male (% male)**	8 (38%)	16 (34%)	15 (56%)	16 (40%)	30 (39%)	0.338	3 (27%)	16 (57%)	6 (32%)	2 (29%)	19 (41%)	25 (39%)	0.141
**APOEε4 carriers**	7/21 (33%)	5/47 (11%)^a^	7/26 (27%)	22/40 (55%)	51/76 (67%)^a^	**< 0.001**	1/10 (10%)	4/24 (17%)	3/18 (17%)	3/7 (43%)	24/38 (63%)^a^	39/62 (61%)^a^	**< 0.02**
**Level of education, years**	11.5 (2.4)	10.7 (2.2)	11.0 (2.1)	10.0 (2.5)	10.6 (2.4)	**0.299**	10.4 (4.9)	8.5 (4.6)	7.75 (4.7)	11.4 (4.0)	8.4 (4.6)	7.7 (3.6)	**0.0267**
**MMSE score**	27.14 (2.45)	24.00 (4.01)^a^	23.85 (4.87)^a^	23.82 (3.84)^a^	19.09 (5.74)^a^	**< 0.0001**	28.4 (2.3)	25.6 (2.7)	22.2 (5.8)^a^	28.7 (1.5)	23.3 (3.9)^a^	20.4 (4.8)^a^	**< 0.0001**
**Core biomarkers Lumipulse**													
**CSF Aβ**_**1–42**_	1060.95 (245.23)	1090.66 (468.75)	1027.78 (354.02)	553.00 (209.36)^a^	497.88 (155.27)^a^	**< 0.0001**	1186 (357)	1164 (549)	986 (449)	634 (206)^a^	556 (178)^a^	522 (153)^a^	**< 0.0001**
**CSF Aβ**_**1–42/40**_	0.093 (0.010)	0.088 (0.012)	0.09 (0.01)	0.044 (0.009)^a^	0.042 (0.009)^a^	**< 0.0001**	0.091 (0.017)	0.0879 (0.012)	0.091 (0.017)	0.049 (0.008)^a^	0.043 (0.009)^a^	0.043 (0.008)^a^	**< 0.0001**
**CSF pTau, pg/mL**	32.25 (8.16)	40.97 (20.81)	33.59 (10.50)	94.80 (47.31)^a^	113.53 (60.02)^a^	**< 0.0001**	56.8 (31.4)	50.2 (30.9)	41.0 (24.4)	59.1 (14.3)	112 (59.6)^a^	121.4 (57.9)^a^	**< 0.0001**
**CSF t-tau, pg/mL**	236.62 (63.69)	333,085 (172.630)	362.26 (341.90)	594.83 (285.45)^a^	722.01 (393.87)^a^	**< 0.0001**	307 (141)	357 (230)	311 (166)	407 (107)	669 (323)^a^	759 (342)^a^	**< 0.0001**
**Biomarker profile**													
**Normal profile**	21 (100%)	39 (83%)	25 (92%)	0 (0%)	0 (0%)		9 (82%%)	24 (86%)	17 (89%)	0 (0%)	0 (0%)	0 (0%)	
**AD continuum**	0 (0%)	0 (0%)	0 (0%)	40 (100%)	77 (100%)		0 (0%)	0 (0%)	0 (0%)	7 (100%)	46 (100%)	64 (100%)	
*A+T−*	/	/	/	7 (18%)	10 (13%)		/	/	/	6 (86%)	11 (24%)	3 (5%)	
*A+T+*	/	/	/	33 (82%)	67 (87%)		/	/	/	1 (14%)	35 (76%)	61 (95%)	
**SNAP**	0 (0%)	8 (17%)	2 (8%)	0 (0%)	0 (0%)		2 (18%)	4 (14%)	2 (11%)	0 (0%)	0 (0%)	0 (0%)	
**CSF p-tau biomarkers**													
**CSF N-term p-tau181, pg/mL**	224.72 (66.33)	300.708 (245.52)	242.23 (110.58)	965.91 (628.08) ^a^	1223.94 (802.66)^a^	**< 0.0001**	427 (110)	410 (164)	426 (168)	494 (129)	771 (257)^a^	871 (343)^a^	**< 0.0001**
**CSF N-term p-tau217, pg/mL**	1.84 (0.94)	2.90 (2.50)	2.14 (1.67)	13.88 (9.48)^a^	16.62 (9.74)^a^	**< 0.0001**	N/A	N/A	N/A	N/A	N/A	N/A	N/A
**CSF N-term p-tau231, pg/mL**	271.93 (64.74)	325.05 (148.44)	286.62 (114.72)	657.64 (205.53)^a^	800.58 (640.95)^a^	**< 0.0001**	313 (211)	348 (201)	331 (236)	541 (144)	788 (399)^a^	893 (469)^a^	**< 0.0001**
**CSF N-term p-tau235, pg/mL**	11.19 (2.94)	12.74 (5.67)	11.56 (4.30)	24.75 (8.11)^a^	28.03 (11.52)^a^	**< 0.0001**	17.6 (8.10)	18.5 (11.3)	17.4 (7.23)	23.1 (3.65)	34.8 (12.9)^a^	41.6 (20.0)^a^	**< 0.0001**

Data is shown as mean (SD) or *n* (%), as appropriate. We used Kruskal-Wallis test to compare age between groups and Pearson’s chi-square to compare sex and APOE ε4 frequencies between groups. Fluid biomarkers levels and MMSE were compared with a one-way ANOVA adjusted by age and sex. ^a^*P* < 0.0001 versus controls. ^a^*P* < 0.05 versus CU Aβ−

*Aβ*_*1-42*_ β-amyloid 42, *Aβ*_*1-40*_ β-amyloid 40, *CSF* Cerebrospinal fluid, *CU* Cognitively unimpaired, *MCI* Mild cognitive impairment, *MMSE* Mini-Mental State Examination, *N-term* N-terminal, *p-tau181* Tau phosphorylated at threonine 181, *p-tau217* Tau phosphorylated at threonine 217, *p-tau231* Tau phosphorylated at threonine 231, *p-tau235* Tau phosphorylated at threonine 181, *t-tau* Total tau

### CSF p-tau235 across syndromic and CSF Aβ-positive and CSF Aβ-negative groups

**Fig. 1:**
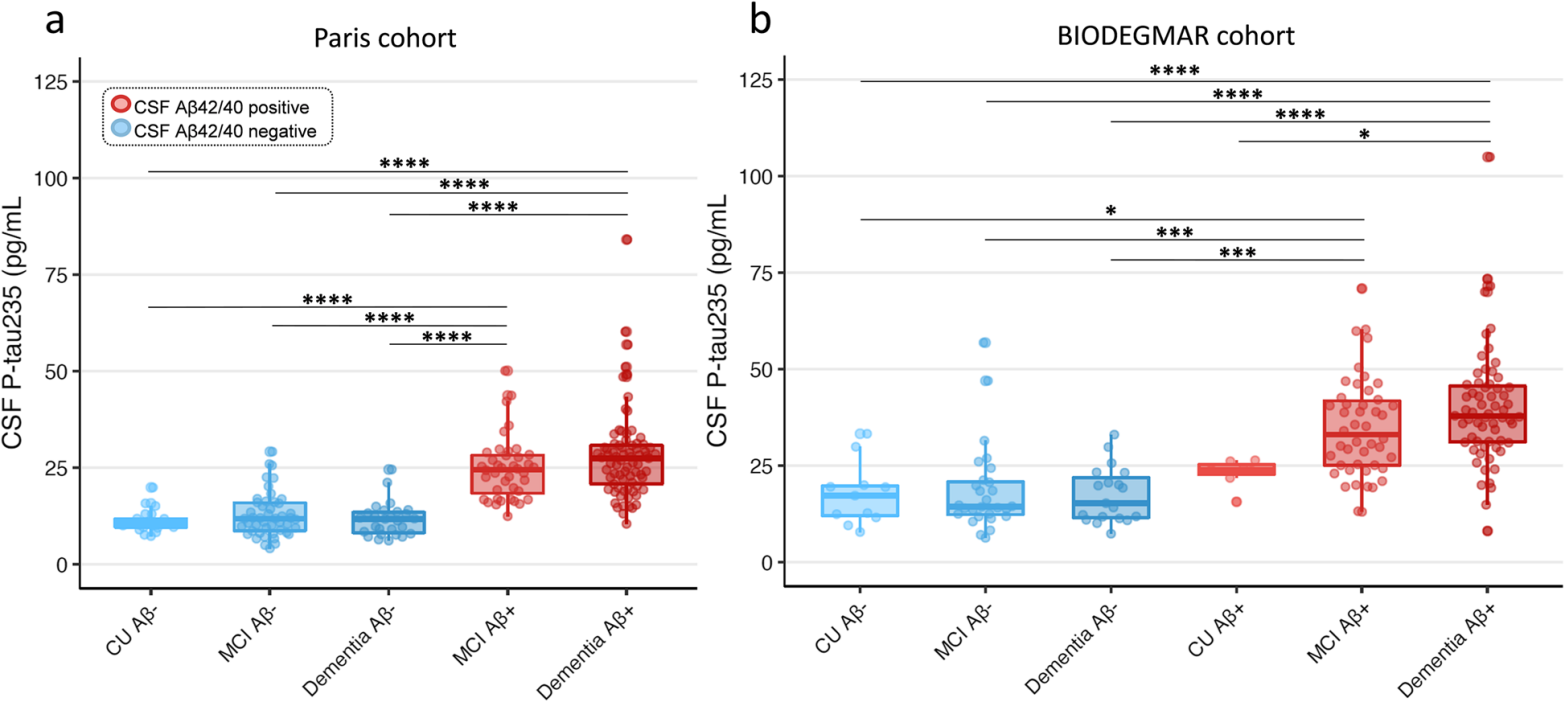
CSF levels of p-tau235 across syndromic groups stratified by CSF amyloidosis. **a** In the Paris cohort, CSF p-tau235 was significantly increased in both dementia Aβ+ and MCI Aβ+ when compared with CU Aβ−, MCI Aβ− and dementia Aβ−. **b** In the BIODEGMAR cohort, CSF p-tau235 was significantly higher in both dementia Aβ+ and MCI Aβ+ when compared with CU Aβ−, MCI Aβ− and dementia Aβ−. Additionally, CSF p-tau235 was significantly increased in dementia Aβ+ when compared with CU Aβ+. *Data information*: Boxplots show the median, IQR, and all the participants colour-coded based on the presence (red) or absence (blue) of CSF amyloidosis measured with Lumipulse CSF Aβ Aβ_1–42/40_. *P*-values were determined using one-way ANOVA adjusted by age and sex, followed by Bonferroni-corrected post hoc comparison (*P <0.05, **P <0.01, ***P <0.001, ****P <0.0001)

All comparisons of multiple groups were adjusted for age and sex. In the Paris cohort, CSF p-tau235 was significantly higher in MCI Aβ+ and dementia Aβ+ when compared with CU Aβ−, MCI Aβ− and dementia Aβ− groups (all *P*_*ADJ*_ <0.0001) (Fig. 1a). Similarly, in the BIODEGMAR cohort, CSF p-tau235 was significantly increased in MCI Aβ+ and dementia Aβ+ when compared with CU Aβ−, MCI Aβ− and dementia Aβ− (all *P*_*ADJ*_ <0.001) (Fig. 1b). CSF p-tau235 was also significantly higher in dementia Aβ+ participants when compared with CU Aβ+ (despite this group being only comprised by seven subjects, *P*_*ADJ*_ =0.038), but no statistical differences were found between MCI Aβ+ and CU Aβ+. No significant differences were found between CU Aβ− and CU Aβ+, likely due to small sample sizes (eleven and seven cases, respectively), although higher CSF p-tau235 levels in CU Aβ+ were noticeable (Fig. 1b).

### CSF p-tau235 discriminatory accuracy identifying CSF amyloidosis in syndromic groups

**Fig. 2:**
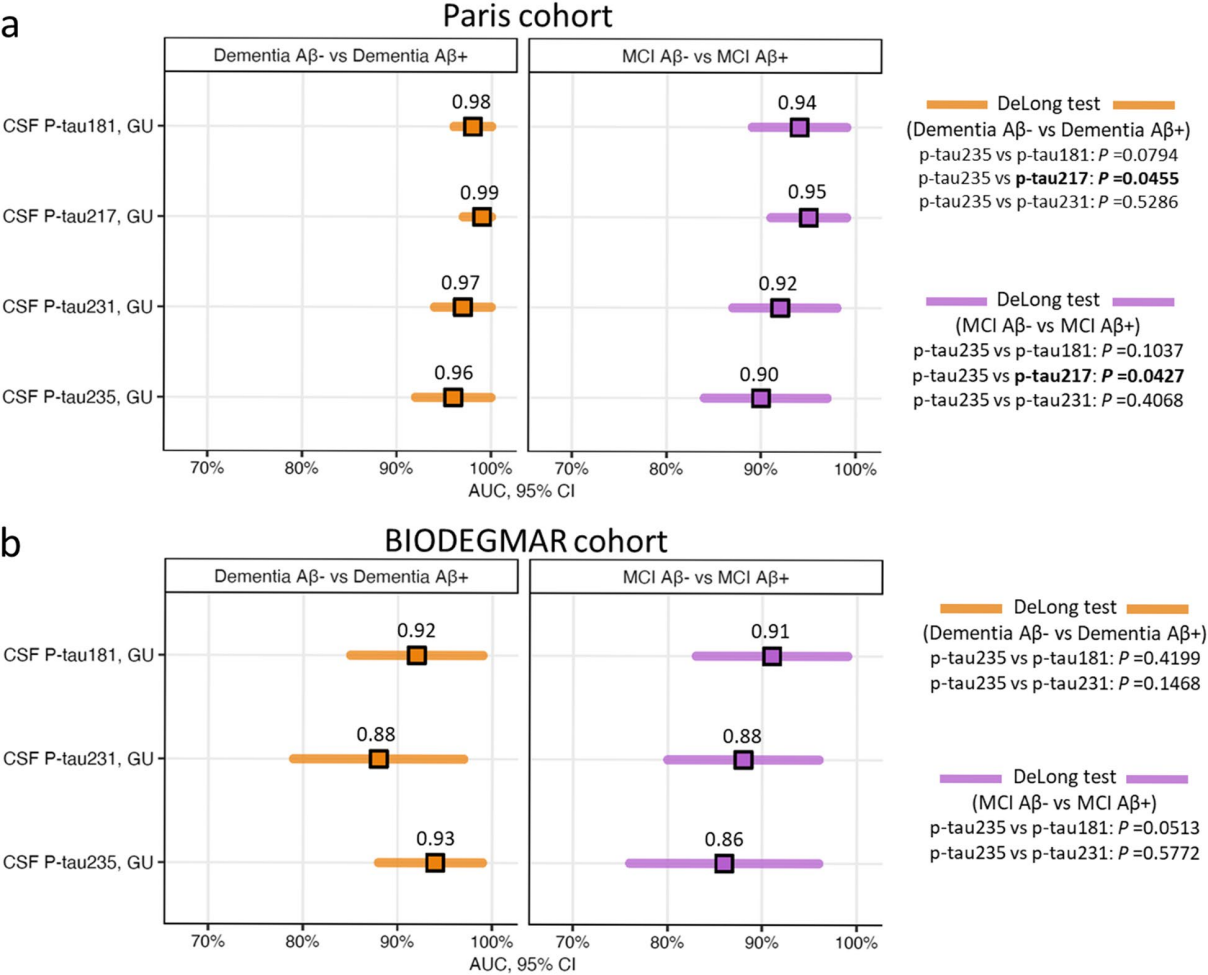
Diagnostic performance of CSF p-tau235 when discriminating syndromic groups stratified by CSF amyloidosis. Both in **a** the Paris cohort and **b** the BIODEGMAR cohort CSF p-tau235 displayed high accuracies differentiating dementia Aβ+ from dementia Aβ− and MCI Aβ+ from MCI Aβ−, similar to CSF p-tau181 and p-tau231, and lower than CSF p-tau217. *Data information*: forest plots showing AUC values from ROC analysis (CI_95%_ available in Supplementary Table 4). Comparisons of AUC values between CSF p-tau235 and other available CSF p-tau biomarkers were determined using DeLong test (significance is indicated in bold)

We then investigated the discriminatory accuracies of CSF p-tau235 to detect CSF amyloidosis between each of the syndromic groups and compared its performance with other novel N-terminal directed CSF p-tau biomarkers, specifically p-tau181, p-tau217 and p-tau231 (Fig. 2). In the Paris cohort, CSF p-tau235 displayed high accuracies when discriminating dementia Aβ− from dementia Aβ+ (AUC_Dementia Aβ− vs Aβ+_=0.96, CI_95%_=0.90–0.99) and MCI Aβ+ from MCI Aβ− (AUC_MCI Aβ− vs Aβ+_=0.90, CI_95%_=0.82–0.96) (Fig. 2a). In these two scenarios, CSF p-tau235 performance showed no statistical differences when compared with CSF p-tau181 (AUC_DementiaAβ− vs Aβ+_=0.98, CI_95%_=0.93–1.00; AUC_MCI Aβ− vs Aβ+_=0.94; CI_95%_=0.86–0.98) and CSF p-tau231 (AUC_DementiaAβ− vs Aβ+_=0.97, CI_95%_=0.92–0.99, AUC_MCI Aβ− vs Aβ+_=0.92, CI_95%_=0.85–0.97). In contrast, lower accuracies were observed when compared with CSF p-tau217 (AUC_DementiaAβ− vs Aβ+_=0.99, CI_95%_=0.94–1.00; AUC_MCI Aβ− vs Aβ+_=0.95, CI_95%_=0.88–0.99; both *P*˂0.05) (Fig. 2a). In the BIODEGMAR cohort, CSF p-tau235 showed high performance discriminating dementia Aβ+ from dementia Aβ− (AUC_Dementia Aβ− vs Aβ+_=0.93, CI_95%_=0.86–0.98) and MCI Aβ+ from MCI Aβ− (AUC_MCI Aβ− vs Aβ+_=0.86, CI_95%_=0.76–0.93) (Fig. 2b). When compared with other p-tau species, CSF p-tau235 performance discriminating dementia Aβ+ from dementia Aβ− was equal to CSF p-tau181 (AUC_DementiaAβ− vs Aβ+_=0.92, CI_95%_=0.84–0.97) and CSF p-tau231 (AUC_DementiaAβ− vs Aβ+_=0.88, CI_95%_=0.79–0.94). On the other hand, the accuracy of CSF p-tau235 distinguishing MCI Aβ+ from MCI Aβ− approached significance compared with CSF p-tau181 (AUC_MCI Aβ− vs Aβ+_=0.91, CI_95%_=0.82–0.96; *P*=0.0513), while matched CSF p-tau231 (AUC_MCI Aβ− vs Aβ+_=0.88, CI_95%_=0.78–0.94) (Fig. 2b).

### CSF p-tau235 across AT groups

**Fig. 3:**
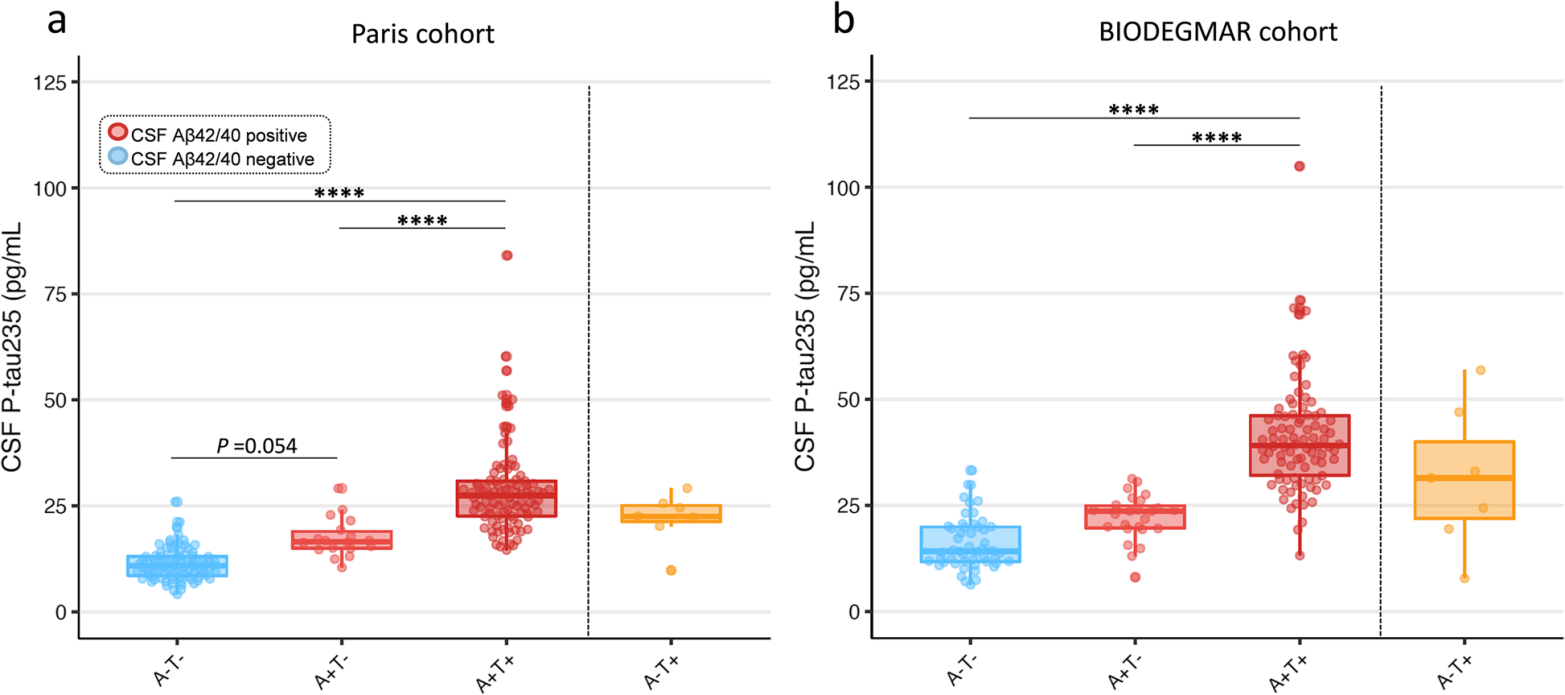
CSF levels of p-tau235 across AT groups. In both **a** the Paris cohort and **b** the BIODEGMAR cohort, CSF p-tau235 followed an increasing trajectory across the AT *continuum*. CSF p-tau235 was significantly increased in A+T+ when compared with A−T− and A+T−, and approached significance between A−T− and A+T− in the Paris cohort. *Data information*: Boxplots show the median, IQR, and all the participants colour-coded based on the presence (red) or absence (blue) of CSF amyloidosis measured with Lumipulse CSF Aβ_1–42/40_. *P*-values were determined using one-way ANOVA adjusted by age and sex, followed by Bonferroni-corrected post hoc comparison (*P <0.05, **P <0.01, ***P <0.001, ****P <0.0001)

All comparisons of multiple groups were adjusted for age and sex. In the Paris cohort, CSF p-tau235 levels progressively increased across the AD *continuum*, that is, from A−T− to A+T− and from A+T− to A+T+ (Fig. 3a). A borderline significant increase in CSF p-tau235 levels was observed between A−T− and A+T− (*P*_*ADJ*_ =0.054). This was followed by a prominent increase between A+T− and A+T+ (*P*_*ADJ*_ <0.0001). CSF p-tau235 concentration was also significantly higher in A+T+ when compared with A−T− (*P*_*ADJ*_ <0.0001) (Fig. 3a). In the BIODEGMAR cohort, CSF p-tau235 also followed an increasing trajectory across the AD *continuum*, although no significant differences were found between A−T− and A+T− subjects when performing multiple comparisons (Fig. 3b). On the other hand, the levels of CSF p-tau235 were significantly higher in A+T+ when compared with A−T− and A+T− (*P*_*ADJ*_ <0.0001 for both) (Fig. 3b). Finally, we stratified patients based on exclusively CSF Aβ, which resulted in CSF p-tau235 being highly increased in A+ cases when compared with A− in both cohorts (*P*<0.0001 for all) (Supplementary Figure 2).

### CSF p-tau235 discriminatory accuracy identifying CSF amyloidosis in AT groups

**Fig. 4:**
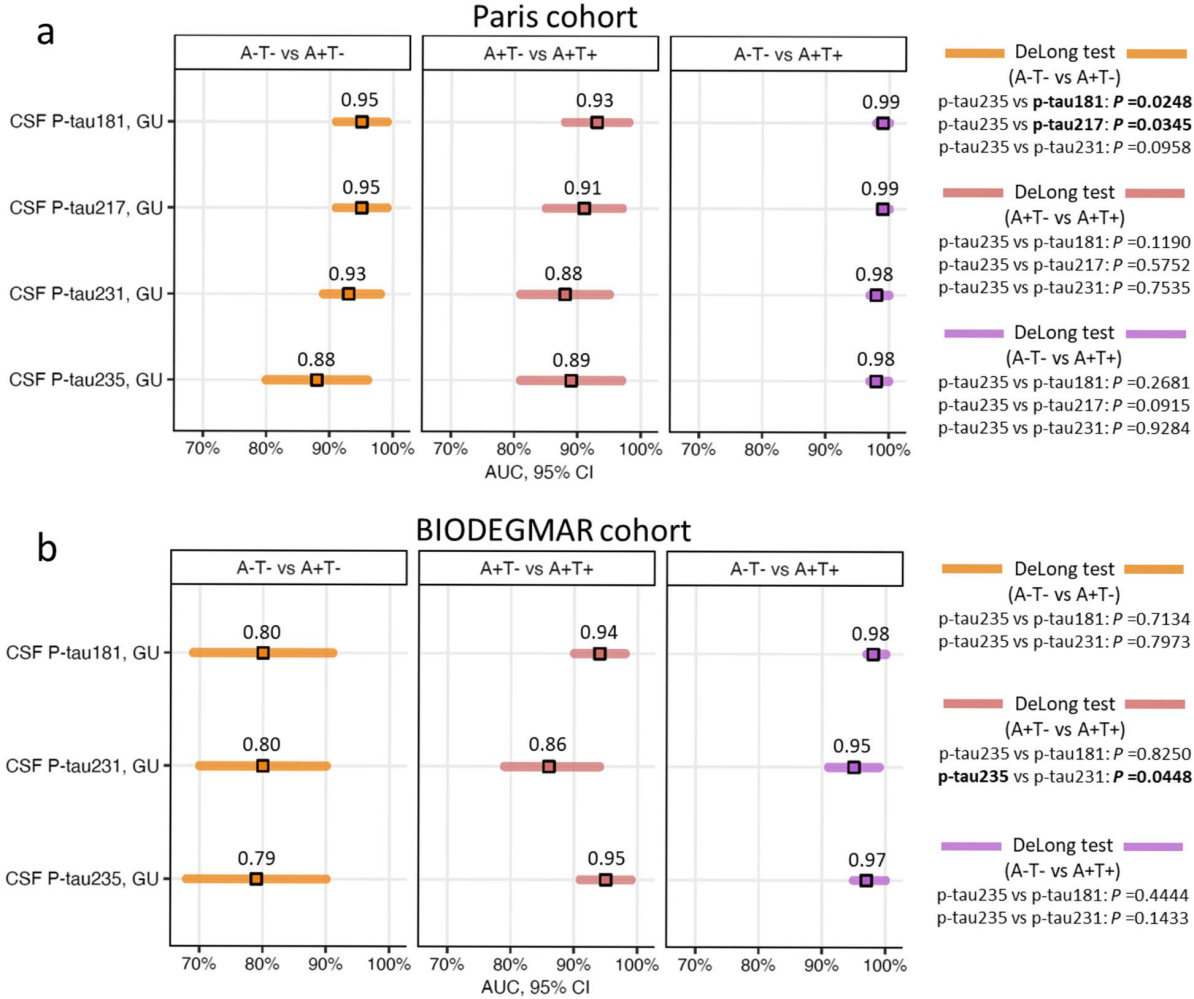
Diagnostic performance of CSF p-tau235 when discriminating AT groups. **a** In the Paris cohort, CSF p-tau235 showed high accuracies in all three scenarios. CSF p-tau235 performance discriminating A−T− from A+T− was lower than CSF p-tau181 and p-tau217, but matched that of CSF p-tau231. When differentiating A+T− from A+T+ and A−T− from A+T+, CSF p-tau235 matched the performance of CSF p-tau181, p-tau217 and p-tau231. **b** In the BIODEGMAR cohort, CSF p-tau235 displayed high accuracies in all three scenarios, equal to those of CSF p-tau181 and p-tau231, slightly outperforming the latter when discriminating A+T− from A+T+. *Data information*: Forest plots showing AUC values from ROC analysis (CI_95%_ available in Supplementary Table 7). Comparisons of AUC values between CSF p-tau235 and other available CSF p-tau biomarkers were determined using DeLong test (significance is indicated in bold)

We then examined the performance of CSF p-tau235 for discriminating the AT groups which comprise the AD *continuum*, that is A−T−, A+T− and A+T+ (Fig. 4). In the Paris cohort, CSF p-tau235 displayed high accuracy discriminating A−T− from A+T− (AUC_A−T− vs A+T−_=0.88, CI_95%_=0.80_−_0.93), similar to CSF p-tau231 (AUC_A−T− vs A+T−_=0.93, CI_95%_=0.87–0.97), but lower than both CSF p-tau181 (AUC_A−T− vs A+T−_=0.95, CI_95%_=0.89–0.98, *P*˂0.05) and CSF p-tau217 (AUC_A−T− vs A+T−_=0.95, CI_95%_=0.89–0.98, *P*˂0.05) (Fig. 4a). CSF p-tau235 showed high accuracy discriminating A+T− from A+T+ participants (AUC_A+T− vs A+T+_=0.89, CI_95%_=0.82–0.94) and A−T− from A+T+ (AUC_A−T− vs A+T+_=0.98, CI_95%_=0.95–1.00). In both scenarios, CSF p-tau235 performance was statistically equal to that of CSF p-tau181 (AUC_A+T− vs A+T+_=0.93, CI_95%_=0.87–0.97; AUC_A−T− vs A+T+_=0.99, CI_95%_=0.97–1.00), CSF p-tau217 (AUC_A+T− vs A+T+_=0.91, CI_95%_=0.84–0.96; AUC_A−T− vs A+T+_=0.99, CI_95%_=0.97–1.00) and CSF p-tau231 (AUC_A+T− vs A+T+_=0.88, CI_95%_=0.81–0.93; AUC_A−T− vs A+T+_=0.98, CI_95%_=0.95–1.00) (Fig. 4a).

In the BIODEGMAR cohort (Fig. 4b), CSF p-tau235 displayed equal accuracies when discriminating A−T− from A+T− (AUC_A−T− vs A+T−_=0.79, CI_95%_=0.68–0.87) as CSF p-tau181 (AUC_A−T− vs A+T−_=0.80, CI_95%_=0.69–0.88) and CSF p-tau231 (AUC_A−T− vs A+T−_=0.80, CI_95%_=0.70–0.88). The accuracy of CSF p-tau235 when discriminating A+T− and A+T+ (AUC_A+T− vs A+T+_=0.95, CI_95%_=0.88–0.98) statistically matched that of CSF p-tau181 (AUC_A+T- vs A+T+_=0.94, CI_95%_=0.88–0.98) and slightly outperformed that of CSF p-tau231 (AUC_A+T− vs A+T+_=0.86, CI_95%_=0.79–0.92, *P*=0.0448). Finally, CSF p-tau235 showed a nearly perfect accuracy discriminating A−T− and A+T+ (AUC_A−T− vs A+T+_=0.97, CI_95%_=0.93–0.99), same as that observed for CSF p-tau181 (AUC_A−T− vs A+T+_=0.98, CI_95%_=0.94–1.00) and CSF p-tau231 (AUC_A−T− vs A+T+_=0.95, CI_95%_=0.90–0.98) (Fig. 4b).

Finally, we evaluated the performance of CSF p-tau235 when discriminating Aβ+ from Aβ− individuals (Supplementary Figure 3, Supplementary Table 8). In the Paris cohort, CSF p-tau235 showed high accuracy distinguishing these two groups (AUC_Aβ− vs Aβ+_= 0.94, CI_95%_=0.90–0.97). When compared with other p-tau species (Supplementary Table 8), CSF p-tau235 performance was significantly lower than CSF p-tau181 (AUC_Aβ− vs Aβ +_=0.97, CI_95%_=0.93–0.99, *P*˂0.05) and CSF p-tau 217 (AUC_Aβ− vs Aβ +_=0.98, CI_95%_=0.95–0.99, *P*˂0.01), but equal to CSF p-tau231 (AUC_Aβ− vs Aβ+_=0.96, CI_95%_=0.92–0.98) (Supplementary Figure 3a). In the BIODEGMAR cohort, CSF p-tau235 (AUC_Aβ− vs Aβ+_ =0.89, CI_95%_=0.83–0.93) matched the performance of both CSF p-tau181 (AUC_Aβ− vs Aβ+_=0.91, CI_95%_=0.85–0.94) and CSF p-tau231 (AUC_Aβ− vs Aβ+_=0.88, CI_95%_=0.82–0.92) (Supplementary Figure 3b).

### CSF p-tau235 association with cognition

In both cohorts, MMSE score was associated with CSF p-tau235 after adjustment on age, sex and level of education (Paris: β=−0.304, *P*_*ADJ*_ <0.0001; BIODEGMAR: β=−0.247, *P*_*ADJ*_ =0.0009). The association remained significant after further adjustment on Aβ_1–42/40_ ratio (Paris Cohort: β=−0.243, *P*_*ADJ*_ =0.0012; BIODEGMAR cohort: β=−0.225, *P*_*ADJ*_ =0.004).

**Fig. 5:**
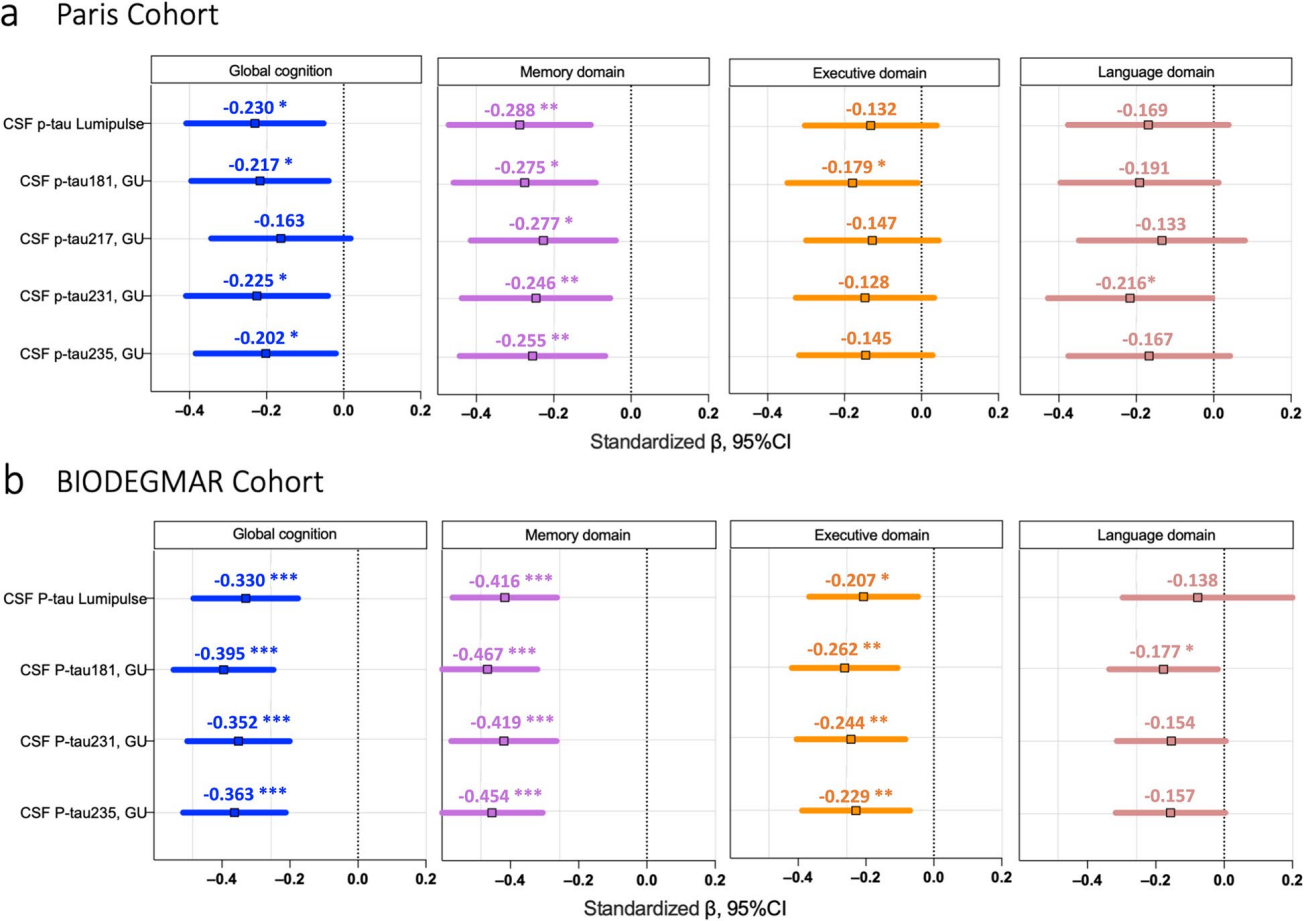
CSF p-tau 235 association with global cognition and specific cognitive domains. Comparison of CSF p-tau235 and other p-tau markers (p-tau181, p-tau217 and p-tau231) for global and domain-specific cognition in **a** the Paris and **b** the BIODEGMAR cohort. *Data information*: Linear regressions were adjusted for age, sex and level of education. Estimates are presented standardized to allow for comparison in between the different p-tau biomarkers. (*P <0.05, **P <0.01, ***P <0.001, ****P <0.0001)

Neuropsychological assessment was available for a subset of patients in the Paris (*n*=136) and BIODEGMAR cohort (*n*=139). The cross-sectional associations of CSF p-tau235 levels and other CSF p-tau measurements with global and domain-specific cognition are shown in Fig. 5, after adjustment on age, sex and level of education. CSF p-tau 235 associated with global cognition in both cohorts (Paris β=−0.202, *P*_*ADJ*_ =0.046; BIODEGMAR β=−0.363, *P*_*ADJ*_ <0.0001), similar to all CSF p-tau measurements but CSF p-tau217. Regarding specific cognitive domains, the strongest association was found with memory impairment, both for CSF p-tau235 and other p-tau species (CSF p-235: Paris β= −0.255, *P*_*ADJ*_ =0.0031; BIODEGMAR: β= −0.454, *P*_*ADJ*_ <0.0001). A weak association was also found with executive domain function but only in BIODEGMAR (β= −0.229, *P*_*ADJ*_ =0.0051).

## Discussion

In this study, we investigated the novel CSF biomarker p-tau235 in two independent memory clinic cohorts and compared its diagnostic performance with other relevant p-tau species, specifically CSF p-tau181, p-tau217 and p-tau231. We found that (i) CSF p-tau235 is strongly associated with CSF amyloidosis, regardless of clinical presentation; (ii) high levels of CSF p-tau235 in cognitively impaired patients are highly indicative of MCI or dementia of the AD type; (iii) the main increase in CSF p-tau235 levels occurs between A+T− and A+T+, irrespective of the presence of symptoms or not; (iv) CSF p-tau235 displayed overall the same diagnostic performance as CSF p-tau181 and p-tau231, but a slightly lower diagnostic performance compared with CSF p-tau217; and (v) CSF p-tau235 levels associate with global and specific-domain cognitive decline.

In a previous study, we described CSF p-tau235 across the AD *continuum* using two research cohorts, demonstrating its high specificity for AD and its comparable performance to other CSF p-tau species [15]. However, these findings cannot be directly translated into the real-world of memory clinics, which is characterized by higher heterogeneity in population (both in social-economic and ethnic terms), clinical presentations and co-pathologies, vastly contrasting with the stringent inclusion/exclusion criteria and detailed pathophysiological characterizations in research cohorts. Thus, the main goal of the present study was to investigate the translatability of previous CSF p-tau235 results into the routine practice of memory clinic. Consistent with our previous findings in selected research cohorts (similarly stratified using Aβ_1–42/40_) [15], CSF p-tau235 was elevated across CSF Aβ-positive groups in both clinical cohorts. CSF p-tau235 was increased in MCI Aβ+ and dementia Aβ+ showing similar levels between the two groups. This aligns well with previous results in a preclinical AD cohort in which CSF p-tau235 measurements seemingly plateau between MCI Aβ+ and AD Aβ+ [15]. Moreover, in the BIODEGMAR cohort, CSF p-tau235 was noticeably higher in CU Aβ+ when compared with CU Aβ−, and despite lacking sufficient statistical power (eleven and seven cases, respectively), it approached significance. Hence, these results from clinical practice are concordant with our previous results in research cohorts, where CSF p-tau235 was increased in Aβ+ subjects both in preclinical and symptomatic AD [15]. Furthermore, these results indicate that high levels of CSF p-tau235 are strongly associated with CSF amyloidosis and highly indicative of MCI or dementia of an AD type. In terms of diagnostic accuracy identifying Aβ+ in MCI and dementia, CSF p-tau235 showed equal performance to that of CSF p-tau181 and p-tau231, but slightly lower than CSF p-tau217. Because these differences were very marginal, with almost fully overlapping 95% confidence intervals, and together with the lack of CSF p-tau217 measurements in the BIODEGMAR cohort to corroborate these results, this should be cautiously interpreted.

CSF p-tau235 followed an increasing trajectory across AT groups, which was characterized by a minor increment between A−T− and A+T−, followed by a prominent increase from A+T− to A+T+. These differences across AT groups are similar to those previously reported in preclinical AD [15], despite the fact that the cohorts analysed here are memory clinic-based, and thus included proportionally more symptomatic cases. Therefore, this suggests that the increase in CSF p-tau235 is more prominent when abnormalities in CSF Aβ and p-tau are established *(*i.e. A+T+ cases), regardless of these cases being symptomatic, as shown here, or asymptomatic, like previously reported [15]. CSF p-tau235 showed high performance discriminating all three AT groups and was, for the most part, comparable to other CSF p-tau biomarkers. When compared with CSF p-tau181, CSF p-tau235 was outperformed when discriminating A-T- from A+T-, but only in the Paris cohort. This may suggest that N-terminal p-tau181 species emerge earlier in response to Aβ pathology than N-terminal p-tau235 species (which in turn start increasing earlier than mid-region p-tau181) [15, 35]. CSF p-tau217 also outperformed CSF p-tau235 in the same scenario (A−T− vs A+T−) in the Paris cohort, which aligns well with previous results indicating that CSF p-tau217 emerges prior to CSF p-tau235 [15]. On the other hand, no differences in performance could be found between CSF p-tau235 and CSF p-tau231, except when discriminating A+T− and A+T+ in the BIODEGMAR cohort, where CSF p-tau235 slightly outperformed CSF p-tau231. The marginal difference observed is likely due to the fact that CSF p-tau231 emerges very early in preclinical stages, and tends to plateau later on the *continuum* AD, thus rendering a lower discrimination between A+T− and A+T+ groups [12]. Finally, CSF p-tau235 showed a high diagnostic accuracy discriminating Aβ− and Aβ+, however, slightly lower than CSF p-tau181 and clearly lower than CSF p-tau217. Despite this, CSF p-tau235 exhibited the same performance as CSF p-tau231 in both the Paris and BIODEGMAR cohorts. In our previous publication, CSF p-tau235 started to increase in A+T− cases; however, the magnitude or extent of this increase was smaller than for other CSF p-tau species, whereas the increase was more pronounced between A+T− and A+T+ [15]. The same was observed here in this study, and the resulting overlap between the levels of CSF p-tau235 in A−T− and A+T−groups rendered the lower performance of this novel biomarker species identifying CSF amyloidosis. Thus, despite its high performance, other p-tau biomarkers emerge earlier than CSF p-tau235 in response to CSF amyloidosis and, therefore, provide a superior performance discriminating A−T− from A+T− and Aβ− from Aβ+.

Higher CSF p-tau235 levels were associated with lower global cognition and memory function in both cohorts, which had not been previously investigated. CSF p-tau181 and CSF p-tau217 have been reported to associate with global cognition and memory impairment cross-sectional and longitudinally, with p-tau217 showing stronger associations with cognitive decline [36]. In our study, CSF p-tau235 performed overall similarly to other p-tau species. We observed modest differences between assays; however, we cannot determine if this reflects differences in the analytical performances of the assays or differences in the biology underlying tau phosphorylation association with cognition.

### Limitations/strengths

The strengths of this study include the use of two independent memory clinic-based cohorts, allowing us to validate and confirm our findings in two different clinical settings. Secondly, both cohorts had clinically validated biomarker measurements available, which enabled the detailed stratification of participants and the subsequent investigation of CSF p-tau235 in syndromic or AT groups. Moreover, the studied cohorts included measurements of other novel p-tau species, specifically N-terminal directed CSF p-tau181, p-tau217 and p-tau231 (all measured in the same analytical platform), making possible a head-to-head comparison between different p-tau residues. However, this study does not go without limitations. CSF p-tau217 measurements were not available in the BIODEGMAR cohort, and therefore, the biomarker comparison with CSF p-tau235 could only be explored in the Paris cohort. Another limitation is that CU Aβ+ cases were limited to the BIODEGMAR cohort, and their sample size was rather small. Considering that CSF p-tau235 increases late during preclinical AD [15], it is expected that in cohorts richer in CU cases (especially A+T−), other CSF p-tau biomarkers that abnormally emerge earlier in the AD *continuum* (such as p-tau231 and p-tau217) [15] would provide a superior performance identifying CSF amyloidosis in asymptomatic individuals. This would subsequently affect the A−T− vs A+T- analysis, where CSF p-tau235 discriminatory accuracy would likely be lower than that of other CSF p-tau biomarkers*.* A limited number of patients in non-AD dementia group also warranted further studies.

## Conclusions

In conclusion, our memory-clinic-based study including two well-characterized cohorts brings further evidence that p-tau235 is a novel and specific CSF biomarker for AD diagnosis, both at MCI and dementia stages. Comparison with other CSF p-tau species, including p-tau181, p-tau217 and p-tau231, supports that CSF p-tau235 should be suitable for use in clinical settings. In addition, significant association with cognitive decline adds an argument to its relevance as a clinical tool to identify and monitor CSF amyloidosis along the whole AD *continuum*. Future studies will aim to elucidate whether CSF p-tau235 can predict cognitive decline in longitudinal samples and attempt to measure p-tau235 in blood samples.

## Supplementary Information

**Additional file 1: Supplementary Figure 1.** CSF levels of p-tau235 across clinical diagnosis. **Supplementary Figure 2.** CSF levels of p-tau235 in Aβ+ and Aβ- cases. **Supplementary Figure 3.** CSF p-tau235 diagnostic performance discriminating Aβ+ from Aβ- cases. **Supplementary Table 1.** Clinical diagnosis included in each syndrome group in Paris cohort. **Supplementary Table 2.** Clinical diagnosis included in each syndrome group in BIODEGMAR cohort. **Supplementary Table 3.** CSF AD biomarkers cut-offs for BIODEGMAR and Paris Cohort. **Supplementary Table 4.** Accuracies of CSF p-tau181, p-tau217, p-tau231 and p-tau235 when identifying CSF amyloidosis in dementia and MCI cases in Paris and BIODEGMAR cohort. **Supplementary Table 5.** Clinical diagnosis included in each AT group in Paris cohort. **Supplementary Table 6.** Clinical diagnosis included in each AT group in BIODEGMAR cohort. **Supplementary Table 7.** Accuracies of CSF p-tau181, p-tau217, p-tau231 and p-tau235 when discriminating AT groups in Paris and BIODEGMAR cohort. **Supplementary Table 8.** Accuracies of CSF p-tau181, p-tau217, p-tau231 and p-tau235 when discriminating Aβ- from Aβ+ in Paris and BIODEGMAR cohort.

## Data Availability

Bulk anonymized data can be shared by request from qualified investigators, providing data transfer is in agreement with EU legislation and decisions by the institutional review board of each participating center.

## Abbreviations

AD: Alzheimer’s disease
AD-MCI: Mild cognitive impairment due to AD
Aβ: Amyloid beta
ANOVA: Analysis of variance
AUC: Area under the curve
Aβ_1–40_: Aβ peptide 1–40
Aβ_1–42_: Aβ peptide 1–42
Aβ_1–42/40_: Ratio Aβ_1–42_/Aβ_1–40_
bvFTD: Behavioural variant frontotemporal dementia
CAA: Cerebral amyloid angiopathy
CBS: Corticobasal syndrome
CJD: Creutzfeldt-Jakob disease
CSF: Cerebrospinal fluid
CU: Cognitively unimpaired
CU Aβ−: Cognitively unimpaired Aβ negative
CU Aβ−: Cognitively unimpaired Aβ positive
CI_95%_: 95% confidence interval
CV: Coefficient of variation
DLB: Dementia with Lewy bodies
FTD: Frontotemporal dementia
IP-MS: Immunoprecipitation-mass spectrometry
MCI: Mild cognitive impairment
MCI Aβ−: Mild cognitive impairment Aβ negative
MCI Aβ+: Mild cognitive impairment Aβ positive
MMSE: Mini-Mental State Examination
MS: Mass spectrometry
NFT: Neurofibrillary tangles
PPA: Primary progressive aphasia
PSP: Progressive supranuclear palsy
p-tau181: Tau phosphorylated at threonine 181
p-tau217: Tau phosphorylated at threonine 217
p-tau231: Tau phosphorylated at threonine 231
p-tau235: Tau phosphorylated at serine 235
ROC: Receiver operating characteristic curve
SCD: Subjective cognitive decline
r_S_: Spearman’s rank correlation coefficient
Simoa: Single molecule array
t-tau: Total tau
ULOQ: Upper limit of quantification
VCID: Vascular contributions to cognitive impairments and dementia
