# Supplementary material for: Clinical performance and head-to-head comparison of CSF p-tau235 with p-tau181, p-tau217 and p-tau231 in two memory clinic cohorts
Source: Alzheimers Res Ther. 2023 Mar 10;15:48. doi: 10.1186/s13195-023-01201-0 (PMC9999575; doi:10.1186/s13195-023-01201-0)
Supplement: Supplementary file 1 — Additional file 1: Supplementary Figure 1. CSF levels of p-tau235 across clinical diagnosis. Supplementary Figure 2. CSF levels of p-tau235 in Aβ+ and Aβ- cases. Supplementary Figure 3. CSF p-tau235 diagnostic performance discriminating Aβ+ from Aβ- cases. Supplementary Table 1. Clinical diagnosis included in each syndrome group in Paris cohort. Supplementary Table 2. Clinical diagnosis included in each syndrome group in BIODEGMAR cohort. Supplementary Table 3. CSF AD biomarkers cut-offs for BIODEGMAR and Paris Cohort. Supplementary Table 4. Accuracies of CSF p-tau181, p-tau217, p-tau231 and p-tau235 when identifying CSF amyloidosis in dementia and MCI cases in Paris and BIODEGMAR cohort. Supplementary Table 5. Clinical diagnosis included in each AT group in Paris cohort. Supplementary Table 6. Clinical diagnosis included in each AT group in BIODEGMAR cohort. Supplementary Table 7. Accuracies of CSF p-tau181, p-tau217, p-tau231 and p-tau235 when discriminating AT groups in Paris and BIODEGMAR cohort. Supplementary Table 8. Accuracies of CSF p-tau181, p-tau217, p-tau231 and p-tau235 when discriminating Aβ- from Aβ+ in Paris and BIODEGMAR cohort. [file 13195_2023_1201_MOESM1_ESM.docx]

**SUPPLEMENTARY**

**Clinical performance and head-to-head comparison of CSF p-tau235 with p-tau181, p-tau217 and p-tau231 in two memory clinic cohorts.**

Juan Lantero-Rodriguez^1*^, Agathe Vrillon^2,3*^, Aida Fernández-Lebrero^4,5,6*^, Paula Ortiz-Romero^4,5^, Anniina Snellman^1,7^, Laia Montoliu-Gaya^1^, Wagner S. Brum^1,8^, Emmanuel Cognat^2,3^, Julien Dumurgier^3^, Albert Puig-Pijoan^5,6,9^, Irene Navalpotro-Gómez^,5,6^, Greta García-Escobar^6^, Thomas K. Karikari^1,10^, Eugeen Vanmechelen^11^, Nicholas J. Ashton^1,12,13,14^, Henrik Zetterberg^1,15,16,17,18,19^, Marc Suárez-Calvet^4,5,6,20^, Claire Paquet^2,3^, Kaj Blennow^1,15^.

**AFFILIATIONS**

^1^Department of Psychiatry & Neurochemistry, Institute of Neuroscience and Physiology, the Sahlgrenska Academy at the University of Gothenburg, Mölndal, Sweden.

^2^Université de Paris, Institut national de la santé et de la recherche médicale U1144 Optimisation Thérapeutique en Neuropsychopharmacologie, Paris, France.

^3^Centre de Neurologie Cognitive, Groupe Hospitalo Universitaire Assistance Publique Hôpitaux de Paris Nord Hôpital Lariboisière Fernand-Widal, Paris, France.

^4^Barcelonaβeta Brain Research Center (BBRC), Pasqual Maragall Foundation. Barcelona, Spain.

^5^IMIM (Hospital del Mar Medical Research Institute), Barcelona, Spain.

^6^Cognitive Decline and Movement Disorders Unit, Neurology Department, Hospital del Mar, Barcelona, Spain.

^7^Turku PET Centre, University of Turku, Turku University Hospital, Turku, Finland.

^8^Graduate Program in Biological Sciences: Biochemistry, Universidade Federal do Rio Grande do Sul (UFRGS), Porto Alegre, Brazil.

^9^Department of Medicine, Universitat Autònoma de Barcelona, Barcelona, Spain.

^10^Department of Psychiatry, School of Medicine, University of Pittsburgh, Pittsburgh, PA, USA.

^11^ADx NeuroSciences, Technologiepark 94, Ghent, Belgium.

^12^Centre for Age-Related Medicine, Stavanger University Hospital, Stavanger, Norway.

^13^Department of Old Age Psychiatry, Maurice Wohl Clinical Neuroscience Institute, King’s College London, London, UK.

^14^NIHR Biomedical Research Centre for Mental Health & Biomedical Research Unit for Dementia at South London & Maudsley NHS Foundation, London, UK.

^15^Clinical Neurochemistry Laboratory, Sahlgrenska University Hospital, Mölndal, Sweden.

^16^Department of Neurodegenerative Disease, Queen Square Institute of Neurology, University College London, London, UK.

^17^UK Dementia Research Institute, University College London, London, UK.

^18^Hong Kong Center for Neurodegenerative Diseases, Hong Kong, China.

^19^Wisconsin Alzheimer’s Disease Research Center, University of Wisconsin School of Medicine and Public Health, University of Wisconsin-Madison, Madison, WI, USA.

^20^Centro de Investigación Biomédica en Red de Fragilidad y Envejecimiento Saludable (CIBERFES), Madrid, Spain.

*Contributed equally as first authors

Corresponding author. Juan Lantero-Rodriguez, MSc, PhD. Department of Psychiatry and Neurochemistry, Institute of Neuroscience and Physiology, The Sahlgrenska Academy, University of Gothenburg, Gothenburg, Sweden.

Email: juan.rodriguez.2@gu.se

**TABLE OF CONTENT**

**1. SUPPLEMENTARY FIGURES**

**Supplementary Figure 1.** CSF levels of p-tau235 across clinical diagnosis.

**Supplementary Figure 2.** CSF levels of p-tau235 in Aβ+ and Aβ- cases.

**Supplementary Figure 3.** CSF p-tau235 diagnostic performance discriminating Aβ+ from Aβ- cases.

**2. SUPPLEMENTARY TABLES**

**Supplementary Table 1.** Clinical diagnosis included in each syndrome group in Paris cohort.

**Supplementary Table 2.** Clinical diagnosis included in each syndrome group in BIODEGMAR cohort.

**Supplementary Table 3.** CSF AD biomarkers cut-offs for BIODEGMAR and Paris Cohort

**Supplementary Table 4.** Accuracies of CSF p-tau181, p-tau217, p-tau231 and p-tau235 when identifying CSF amyloidosis in dementia and MCI cases in Paris and BIODEGMAR cohort.

**Supplementary Table 5.** Clinical diagnosis included in each AT group in Paris cohort.

**Supplementary Table 6.** Clinical diagnosis included in each AT group in BIODEGMAR cohort.

**Supplementary Table 7.** Accuracies of CSF p-tau181, p-tau217, p-tau231 and p-tau235 when discriminating AT groups in Paris and BIODEGMAR cohort.

**Supplementary Table 8.** Accuracies of CSF p-tau181, p-tau217, p-tau231 and p-tau235 when discriminating Aβ- from Aβ+ in Paris and BIODEGMAR cohort.

**1. SUPPLEMENTARY FIGURES**

**
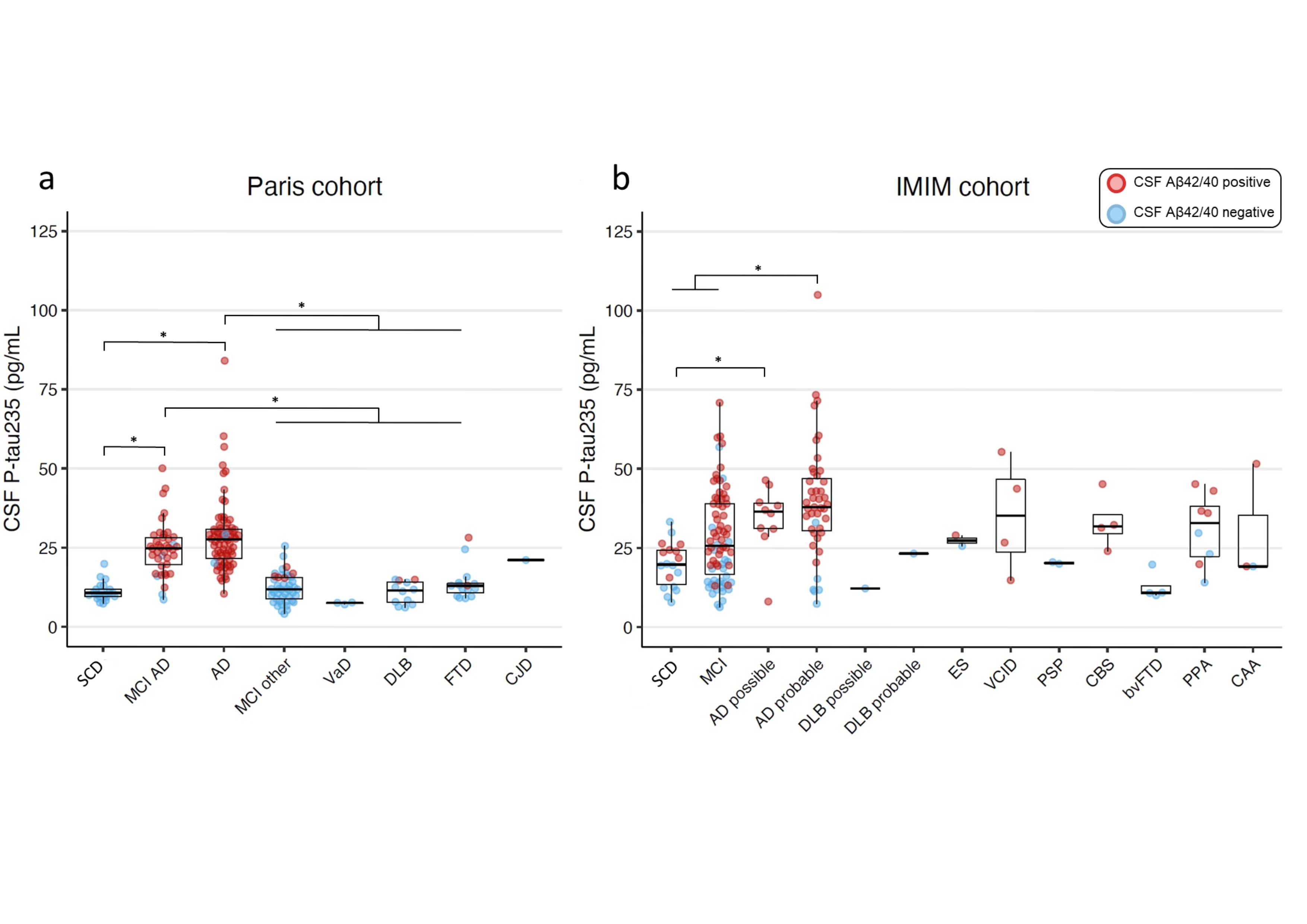
**

**Supplementary Figure 1. CSF levels of p-tau235 across clinical diagnosis in (a) in Paris cohort** **and (b) BIODEGMAR cohort**. *Data* *information*: Boxplots show the median, IQR, and all the participants colour-coded based on the presence (red) or absence (blue) of CSF amyloidosis measured with Lumipulse CSF Aβ Aβ_1-42/40_. *P*-values were determined using one-way ANOVA adjusted by age and sex, followed by Bonferroni-corrected post-hoc comparison (**P* <0.05, ***P* <0.01, ****P* <0.001, *****P* <0.0001). Abbreviations: AD, Alzheimer’s disease; AD-MCI, mild cognitive impairment due to AD; bvFTD, behavioural variant frontotemporal dementia; CAA, cerebral amyloid angiopathy; CBS, corticobasal syndrome; CJD, Creutzfeldt-Jakob disease; DLB, dementia with Lewy Bodies; ES, extrapyramidal syndrome; FTD, frontotemporal dementia; MCI, mild cognitive impairment; PPA, primary progressive aphasia; PSP, progressive supranuclear palsy; SCD, subjective cognitive decline; VCID, vascular contributions to cognitive impairment and dementia.

**
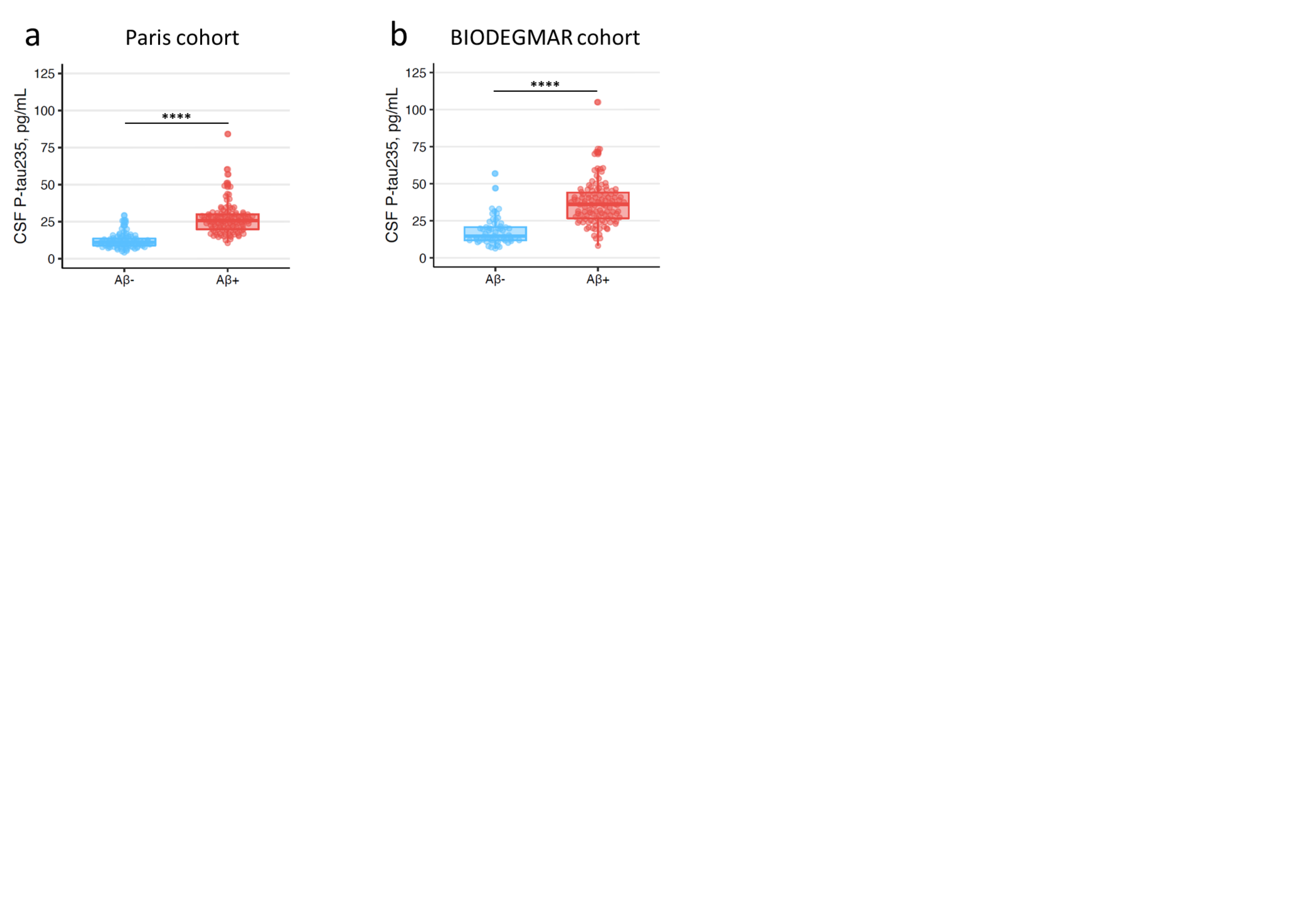
Supplementary Figure 2. CSF levels of p-tau235 in Aβ+ and Aβ- cases.** (**a**) In Paris cohort, CSF p-tau235 was highly increased in Aβ+ when compared with Aβ- cases. (**b**) In IMIM cohort, CSF p-tau235 levels were significantly increased in Aβ+ when compared with Aβ- participants. *Data information*: Boxplots show the median, IQR, and all the participants colour-coded based on the presence (red) or absence (blue) of CSF amyloidosis measured with Lumipulse CSF Aβ Aβ_1-42/40_. *P*-values were determined using Mann-Whitney U test (*****P* < 0.0001).

**
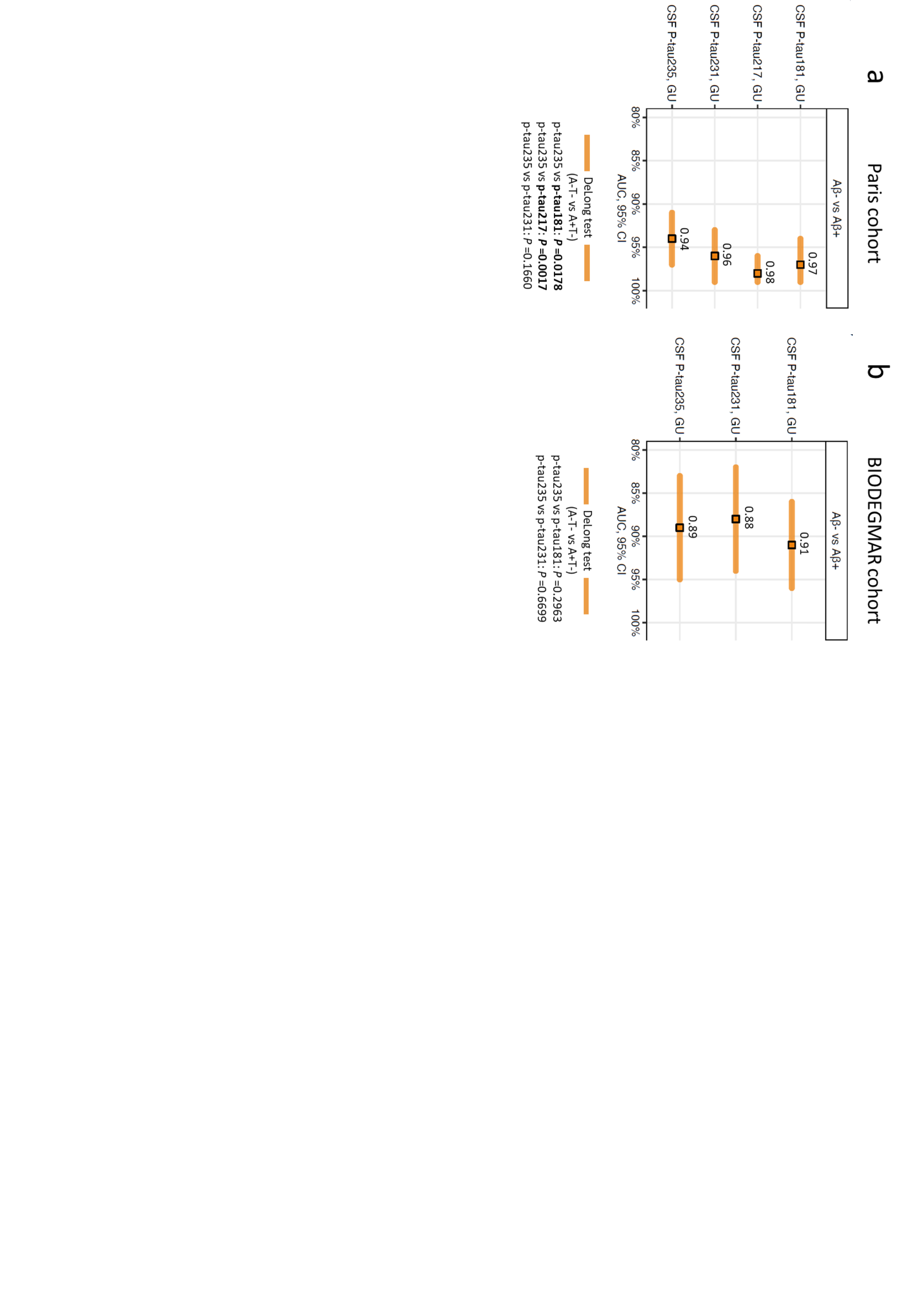
**

**Supplementary Figure 3. CSF p-tau235 diagnostic performance discriminating Aβ+ from Aβ- cases.** (**a**) CSF p-tau235 displayed high diagnostic accuracy discriminating Aβ+ from Aβ- in Paris cohort, statistically equal to that of CSF p-tau231 but lower than CSF p-tau181 and p-tau217. (**b**) CSF p-tau235 showed high AUC values differentiating Aβ+ from Aβ- in IMIM cohort, statistically equal to that of CSF p-tau181 and p-tau231. *Data information*: forest plots showing AUC values from ROC analysis (CI_95%_ available in Supplementary Table 7). Comparisons of AUC values between CSF p-tau235 and other available CSF p-tau biomarkers were determined using DeLong test (significance is indicate in bold).

**2. SUPPLEMENTARY TABLES**

| **Syndrome groups (Paris cohort)** | | | | |
| --- | --- | --- | --- | --- |
| **CU Aβ-**  **(n=21)** | **MCI Aβ-**  **(n=47)** | **Dementia Aβ-**  **(n=27)** | **MCI Aβ+**  **(n=40)** | **Dementia Aβ+ (n=77)** |
| SCD (n=21) | AD (n=2)  AD-MCI (n=5)  Non-AD MCI (n=40) | CJD (n=1)  DLB (n=10)  FTD (n=13)  VaD (n=3) | AD-MCI (n=35)  Non-AD MCI (n=5) | AD (n=73)  DLB (n=2)  FTD (n=2) |

**Supplementary Table 1. Clinical diagnosis included in each syndrome group in Paris cohort.** Abbreviations: AD, Alzheimer’s disease; AD-MCI, mild cognitive impairment due to AD; CJD, Creutzfeldt-Jakob disease; DLB, dementia with Lewy Bodies; FTD, frontotemporal dementia; SCD, subjective cognitive decline; VaD, vascular dementia.

| **Syndrome groups (BIODEGMAR cohort)** | | | | | |
| --- | --- | --- | --- | --- | --- |
| **CU Aβ-**  **(n=11)** | **MCI Aβ-**  **(n=28)** | **Dementia Aβ-**  **(n=19)** | **CU Aβ+**  **(n=7)** | **MCI Aβ+**  **(n=46)** | **Dementia Aβ+ (n=64)** |
| SCD (n=11) | MCI (n=28) | AD probable (n=6)  bvFTD (n=4)  CAA (n=1)  DLB (n=2)  ES (n=1)  PPA (n=3)  PSP (n=2) | SCD (n=7) | MCI (n=46) | ES (n=1)  AD probable (n=37)  AD possible (n=11)  CAA (n=2)  CBS (n=4)  PPA (n=5)  VCID (n=4) |

**Supplementary Table 2. Clinical diagnosis included in each syndrome group in BIODEGMAR cohort.** Abbreviations: AD, Alzheimer’s disease; bvFTD, behavioural variant frontotemporal dementia; CAA, cerebral amyloid angiopathy; CBS, corticobasal syndrome; DLB, dementia with Lewy Bodies, ES, extrapyramidal syndrome; MCI, mild cognitive impairment; PPA, primary progressive aphasia; PSP, progressive supranuclear palsy; SCD, subjective cognitive decline; VCID, vascular contributions to cognitive impairment and dementia.

| **Paris cohort** | **Lumipulse®, normal ranges^a^** |
| --- | --- |
| CSF **Aβ42/ Aβ40 ratio** | >0.061 |
| CSF p-tau181 (pg/ml) | < 0.61 |
| CSF t-tau (pg/ml) | <450 |
| **BIODEGMAR cohort** | **Lumipulse®, normal ranges^b^** |
| CSF **Aβ42/ Aβ40 ratio** | >0.062 |
| CSF p-tau181 (pg/ml) | < 69.85 |
| CSF t-tau (pg/ml) | < 522 |

**Supplementary Table 3. CSF AD biomarkers cut-offs for BIODEGMAR and Paris Cohort**

^a^ Cut offs used for Paris cohort are detailed in Karikari TK, Emeršič A, Vrillon A, et al . *Head-to-head comparison of clinical performance of CSF phospho-tau T181 and T217 biomarkers for Alzheimer's disease diagnosis*. Alzheimer’s & Dementia 2021.

**^b^** Validation of the cut-offs in the BIODEGMAR cohort is reported in Puig-Pijoan A, et al. *The CORCOBIA study: Cut-off points of Alzheimer's disease CSF biomarkers in a clinical cohort*. Neurologia (English Edition). 2022.

| **Paris cohort** | **Dementia Aβ- vs Dementia Aβ+** | **MCI Aβ- vs MCI Aβ+** |
| --- | --- | --- |
|  | **AUC (95% CI)** | |
| CSF p-tau181 | 0.98 (0.93–1.00) | 0.94 (0.86–0.98) |
| CSF p-tau217 | 0.99 (0.94–1.00) | 0.95 (0.88–0.99) |
| CSF p-tau231 | 0.97 (0.92–0.99) | 0.92 (0.85-0.97) |
| CSF p-tau235 | 0.96 (0.90-0.99) | 0.90 (0.82-0.96) |
| **BIODEGMAR cohort** | **Dementia Aβ- vs Dementia Aβ+** | **MCI Aβ- vs MCI Aβ+** |
|  | **AUC (95% CI)** | |
| CSF p-tau181 | 0.92 (0.84-0.97) | 0.91 (0.82-0.96) |
| CSF p-tau217 | N/A | N/A |
| CSF p-tau231 | 0.88 (0.79-0.94) | 0.88 (0.78-0.94) |
| CSF p-tau235 | 0.93 (0.86-0.98) | 0.86 (0.76-0.93) |

**Supplementary Table 4. Accuracies of CSF p-tau181, p-tau217, p-tau231 and p-tau235 when identifying CSF amyloidosis in dementia and MCI cases in Paris and BIODEGMAR cohort.**

| **AT groups (Paris cohort)** | | | |
| --- | --- | --- | --- |
| **A-T-**  **(n=88)** | **A+T-**  **(n=18)** | **A+T+**  **(n=99)** | **A-T+**  **(n=7)** |
| AD-MCI (n=4)  CJD (n=1)  DLB (n=10)  FTD (n=11)  Non-AD MCI (n=38)  SCD (n=21)  VaD (n=3) | AD (n=8)  DLB (n=2)  FTD (n=1)  AD-MCI (n=6)  Non-AD MCI (n=1) | AD (n=65)  AD-MCI (n=29)  FTD (n=1)  Non-AD MCI (n=4) | AD (n=2)  AD-MCI (n=1)  FTD (n=2)  Non-AD MCI (n=2) |

**Supplementary Table 5. Clinical diagnosis included in each AT group in Paris cohort.** Abbreviations: AD, Alzheimer’s disease; AD-MCI, mild cognitive impairment due to AD; CJD, Creutzfeldt-Jakob disease; DLB, dementia with Lewy Bodies, FTD, frontotemporal dementia; SCD, subjective cognitive decline; VaD, vascular dementia.

| **AT groups (BIODEGMAR cohort)** | | | |
| --- | --- | --- | --- |
| **A-T-**  **(n=51)** | **A+T-**  **(n=26)** | **A+T+**  **(n=91)** | **A-T+**  **(n=7)** |
| AD probable (n=5)  bvFTD (n=4)  CAA (n=1)  DLB (n=2)  ES (n=1)  MCI (n=24)  SCD (n=9)  PPA (n=3)  PSP (n=2) | AD possible (n=2)  AD probable (n=2)  CBS (n=1)  ES (n=1)  MCI (n=11)  PPA (n=1)  SCD (n=6)  VCID (n=2) | AD possible (n=9)  AD probable (n=35)  CAA (n=2)  CBS (n=3)  MCI (n=35)  PPA (n=4)  SCD (n=1)  VCID (n=2) | AD probable (n=1)  MCI (n=4)  SCD (n=2) |

**Supplementary Table 6. Clinical diagnosis included in each AT group in BIODEGMAR cohort.** Abbreviations: AD, Alzheimer’s disease; bvFTD, behavioural variant frontotemporal dementia; CAA, cerebral amyloid angiopathy; CBS, corticobasal syndrome; DLB, dementia with Lewy Bodies, ES, extrapyramidal syndrome, MCI, mild cognitive impairment; PPA, primary progressive aphasia; PSP, progressive supranuclear palsy, SCD, subjective cognitive decline; VCID, vascular contributions to cognitive impairment and dementia.

| **Paris cohort** | **A-T- vs A+T-** | **A+T- vs A+T+** | **A-T- vs A+T+** |
| --- | --- | --- | --- |
|  | **AUC (95% CI)** | | |
| CSF p-tau181 | 0.95 (0.89-0.98) | 0.93 (0.87-0.97) | 0.99 (0.97-1.00) |
| CSF p-tau217 | 0.95 (0.89-0.98) | 0.91 (0.84-0.96) | 0.99 (0.97-1.00) |
| CSF p-tau231 | 0.93 (0.87-0.97) | 0.88 (0.81-0.93) | 0.98 (0.95-1.00) |
| CSF p-tau235 | 0.88 (0.80-0.93) | 0.89 (0.82-0.94) | 0.98 (0.95-1.00) |
| **BIODEGMAR cohort** | **A-T- vs A+T-** | **A+T- vs A+T+** | **A-T- vs A+T+** |
|  | **AUC (95% CI)** | | |
| CSF p-tau181 | 0.80 (0.69-0.88) | 0.94 (0.88-0.98) | 0.98 (0.94-1.00) |
| CSF p-tau217 | N/A | N/A | N/A |
| CSF p-tau231 | 0.80 (0.70-0.88) | 0.86 (0.79-0.92) | 0.95 (0.90-0.98) |
| CSF p-tau235 | 0.79 (0.68-0.87) | 0.95 (0.88-0.98) | 0.97 (0.93-0.99) |

**Supplementary Table 7. Accuracies of CSF p-tau181, p-tau217, p-tau231 and p-tau235 when discriminating AT groups in Paris and BIODEGMAR cohort.**

| **Paris cohort** | **Aβ- vs Aβ+** |
| --- | --- |
|  | **AUC (95% CI)** |
| CSF p-tau181 | 0.97 (0.93-0.99) |
| CSF p-tau217 | 0.98 (0.95-0.99) |
| CSF p-tau231 | 0.96 (0.92-0.98) |
| CSF p-tau235 | 0.94 (0.90-0.97) |
| **BIODEGMAR cohort** | **Aβ- vs Aβ+** |
|  | **AUC (95% CI)** |
| CSF p-tau181 | 0.91 (0.85-0.94) |
| CSF p-tau217 | N/A |
| CSF p-tau231 | 0.88 (0.82-0.92) |
| CSF p-tau235 | 0.89 (0.83-0.93) |

**Supplementary Table 8. Accuracies of CSF p-tau181, p-tau217, p-tau231 and p-tau235 when discriminating in Aβ- from Aβ+ in Paris and BIODEGMAR cohort.**
